# Different Culture Media Affect Proliferation, Surface Epitope Expression, and Differentiation of Ovine MSC

**DOI:** 10.1155/2013/387324

**Published:** 2013-10-21

**Authors:** Carina Adamzyk, Tanja Emonds, Julia Falkenstein, René Tolba, Wilhelm Jahnen-Dechent, Bernd Lethaus, Sabine Neuss

**Affiliations:** ^1^Institute of Laboratory Animal Research, RWTH Aachen University, Pauwelstraße 30, 52074 Aachen, Germany; ^2^Institute of Pathology, RWTH Aachen University, Pauwelstraße 30, 52074 Aachen, Germany; ^3^Helmholtz Institute for Biomedical Engineering, Biointerface Group, RWTH Aachen University, Pauwelstraße 30, 52074 Aachen, Germany; ^4^Institute of Occupational Medicine (Research Center for Bioelectromagnetic Interaction), RWTH Aachen University, Pauwelstraße 30, 52074 Aachen, Germany; ^5^Department of Cranio-Maxillofacial Surgery, RWTH Aachen University, Pauwelstraße 30, 52074 Aachen, Germany

## Abstract

Orthopedic implants including engineered bone tissue are commonly tested in sheep. To avoid rejection of heterologous or xenogeneic cells, autologous cells are preferably used, that is, ovine mesenchymal stem cells (oMSC). Unlike human MSC, ovine MSC are not well studied regarding isolation, expansion, and characterization. Here we investigated the impact of culture media composition on growth characteristics, differentiation, and surface antigen expression of oMSC. The culture media varied in fetal calf serum (FCS) content and in the addition of supplements and/or additional epidermal growth factor (EGF). We found that FCS strongly influenced oMSC proliferation and that specific combinations of supplemental factors (MCDB-201, ITS-plus, dexamethasone, and L-ascorbic acid) determined the expression of surface epitopes. We compared two published protocols for oMSC differentiation towards the osteogenic, adipogenic, and chondrogenic fate and found (i) considerable donor to donor variations, (ii) protocol-dependent variations, and (iii) variations resulting from the preculture medium composition. Our results indicate that the isolation and culture of oMSC in different growth media are highly variable regarding oMSC phenotype and behaviour. Furthermore, variations from donor to donor critically influence growth rate, surface marker expression, and differentiation.

## 1. Introduction

Mesenchymal stem cells (MSC) are a well characterized and highly adaptable cell source for regenerative medicine and tissue engineering. In 1968, Friedenstein and coworkers described for the first time that plating bone marrow cells in serum-containing medium results in the formation of colonies of fibroblast-like adherent cells, capable to differentiate into osteoblasts [1–3]. Today, the role of MSC in clinical applications has been explored in phase I/II clinical trials involving, for example, autoimmune disorders, treatment of acute graft versus host diease (GvHD), and engraftment of hematopoietic cells [4, 5]. Other applications of MSC, especially in combination with biomaterials for the repair of damaged tissues like cartilage and bone, have raised cautious optimism for future therapies. However, novel biomaterials designed for clinical applications are usually tested in several preclinical studies involving animal models. Sheep are a convenient large-animal model for orthopedic research because of their availability, ease of handling and housing, cost, and ethical acceptance [6, 7]. In particular, mature sheep are considered as a valuable model for human bone turnover and remodeling activity, due to the fact that animals of 7–9 years of age show similar bone structure and composition. Furthermore, they possess a body weight comparable to that of adult humans and long bone dimensions enabling the use of human implants [6, 8–10]. Therefore, in orthopaedic research, sheep are frequently employed for critical-size bone defects, which are then treated with different biomaterials combined with (predifferentiated) MSC [8, 9, 11–17]. Despite a considerable number of reports employing oMSC in tissue engineering, characterization of ovine oMSC or proof of multipotency *in vitro *is limited to few studies only [18–23]. In these studies, there is widespread variation in the composition of growth and differentiation media, as well as for analyzing the expression of surface antigens. Growth media for oMSC typically are based on *α*-MEM, DMEM, or DMEM combined with MCDB201. The serum content varies between 2% [24] and 20% [18, 23] of autologous or fetal calf serum (FCS), and other supplements such as ITS-Plus premix, dexamethasone, L-ascorbic acid, or growth factors like epidermal growth factor (EGF) and platelet-derived growth factor (PDGF) may be applied [24]. As a consequence, considerable variances in the cultured oMSC population, and their behaviour in tissue engineering applications are presumed.

For human MSC (hMSC), systematic research has uncovered donor-dependent heterogeneity in proliferation and differentiation [25–28]. Because of varieties in characterization, nomenclature (e.g., bone marrow stromal cells, multipotent adult progenitor cells, and mesenchymal stem cells), and culture media, the *Mesenchymal and Tissue Stem Cell Committee* of the International Society for Cellular Therapy proposed the introduction of a standardization for the phenotypic characterization of hMSC, as stated in their position papers of Horwitz et al. in 2005 [29] and Dominici et al. (2006) [30]. Despite large donor-dependent variations, the differentiation of hMSC towards adipocytes, osteoblasts, and chondrocytes is nowadays standardized according to protocols and media originally published by Pittenger in 1999 [31]. In contrast, no standardized protocols for the characterization and differentiation of oMSC are available so far. 

Here we undertook a systematic study of oMSC cultured in eight different growth media of defined composition. The first medium that was tested is one described several times for the culture of oMSC [21, 22, 32, 33] and consisted of DMEM low glucose containing 10% FCS. The second medium is often used for hMSC culture [34–36] and contained only 2% FCS, but MCDB-201, ITS-Plus, dexamethasone, L-ascorbic acid (summarized abbreviated in the following as “S” for supplements) and 10 ng/mL epidermal growth factor (EGF). Results for differentiation after preculture in the two media were irreproducible and varied strongly for each donor. Furthermore, lots of lipid droplets, indicative for adipogenic differentiation could be observed in the experimental controls, when investigating the trilineage differentiation potential. We therefore decided to break down the oMSC multipotency more systematically and had a detailed view on the expansion, differentiation, and surface marker expression of oMSC, when grown in eight different culture media containing varying FCS and EGF combinations, with or without supplements as described previously. Our results indicate that the proliferation of oMSC is strongly dependent on the FCS content, while EGF and S play only minor roles. Furthermore, the surface epitopes CD73 (5′-ecto-nucleotidase), CD90 (Thy-1), and CD105 (Endoglin) were inducible depending on the FCS but not on the EGF concentration in the culture medium. Finally, the differentiation of oMSC is donor dependent, protocol dependent, and culture medium dependent.

## 2. Materials and Methods

### 2.1. Isolation and Expansion of Ovine MSC

All animal experimentation was carried out according to ILAR and FELASA rules and was approved by an Animal Experimentation Ethics committee appointed by the State of North-Rhine Westfalia. Ovine MSC were isolated out of 40 mL bone marrow aspirate from Rhoen sheep iliac crest that was supplemented with 2 mL 1.107% Di-Sodium-AEDTA (Alleman Pharma, Rimbach, Germany) to prevent coagulation. Mononuclear cells were harvested by Ficoll density gradient centrifugation (density 1.077 g/mL; Biochrom, Berlin, Germany) and subsequently seeded in 25 cm^2^ tissue culture flasks in the growth media detailed as follows. 

The next day, nonadherent ovine cells were removed by medium change. Further medium changes were performed every 3-4 days. At 80–90% confluence, stem cells were trypsinized with stem cell trypsin (Lonza, Walkersville, USA) and reseeded in a density of 500.000 cells per 75 cm^2^ tissue culture flask.

### 2.2. Growth Media

The growth curves and systematic surface antigen analysis were performed with oMSC grown in eight different media containing varying combinations of FCS (F), supplements (S), and EGF. The media composition is summarized in Table 1. 

The growth curve and doubling time were determined by counting the cell numbers after each passage, using a Casy cell counter (Merck, Darmstadt, Germany), for 1–5 passages and using the exponential growth formula:

(1) $$\begin{array}{c} N_{t}=N_{0}•e^{\left(\mu *t\right)}, \end{array}$$

 where *N*_*t*_ is the number of cells at given time point, *N*_0_ is the initial number of cells, *μ* is the growth rate [/day], and *t* is the given time period [days].

The doubling time *T*_*D*_ was calculated by converting the previous formula to (see [37])

(2) $$\begin{array}{c} T_{D}=\frac{\ln2}{\mu }. \end{array}$$

### 2.3. Differentiation

Cell differentiation was induced using cells isolated and cultured in F10 or in F2-S-EGF medium. F10 is analogous to one standard medium used for oMSC in previous studies [22, 33, 38, 39], and F2-S-EGF is a standard medium routinely used for hMSC [34–36, 40].

Differentiation of oMSC was analyzed in passage 2-3, after preculture in F10 or F2-S-EGF. The differentiation was induced by culture for 21 days in induction media according to published protocols for hMSC (protocol 1) [31, 35] and oMSC differentiation (protocol 2) [19, 21]. The composition of the tested media can be taken from Tables 2, 3, and 4; all media contained 2 mM L-glutamine, 100 U/mL penicillin, and 100 *μ*g/mL streptomycin (all PAA, Coelbe, Germany).

Alizarin red staining (40 mM) was used to identify calcium phosphate accumulations. Cell cultures were fixed in 4% formaldehyde for 15 min and washed twice in distilled water. Cells were stained for 20 min, washed in distilled water, and photographed. 

Following protocol 1, adipogenic induction medium was first added after 24 h; afterwards, adipogenic induction and maintenance medium were added alternately. For protocol 2, only adipogenic induction medium was added for 21 days. Media were changed three times a week.

After 30 min of fixation in 50% ice-cold ethanol, 10 min staining in Oil red O staining solution (0.02% w/v dissolved in 3/4 100% methanol and 1/4 1 M NaOH) was used to identify lipid-laden fat cells. Cultures were counterstained with Mayer's Haematoxylin (Sigma, Steinheim, Germany) and photographed.

After 21 days of culture, pellet aggregates were fixed in 4% formaldehyde, paraffin embedded, and histologically stained with safranin red (0.01% w/v in 40% ethanol) for 2 min. After treatment in ascending ethanol series, slides were mounted and photographed. 

### 2.4. Analysis of Surface Epitope Expression by Flow Cytometry

For flow cytometry, cells were trypsinized and approximately 2 × 10^5^ cells per sample were washed twice in PBS. Antibodies frequently used to stain human or ovine MSC (Table 5) were incubated with the cells for 45 min on ice. The samples were either fixed in 1% formalin or measured directly with the FACS-Canto (BD Bioscience, Walkersville, Texas). A decrease of fluorescence by fixation in formalin within 7 days was excluded in previous experiments (data not shown). Unstained cells served as a control for background fluorescence. At least 1 × 10^4^ events were measured per sample. All cells were analyzed in passage 1.

### 2.5. Statistical Analysis

Data analysis was performed using GraphPad Prism 6 software. Comparison between groups was performed via analysis of variance (ANOVA) models with Tukey's multiple comparison tests. Differences with *P* < 0.05 were considered statistically significant. The data were obtained from independent samples (*n* = 3-4) and are expressed as the mean ± standard deviation.

## 3. Results

### 3.1. Morphology

**Figure 1:**
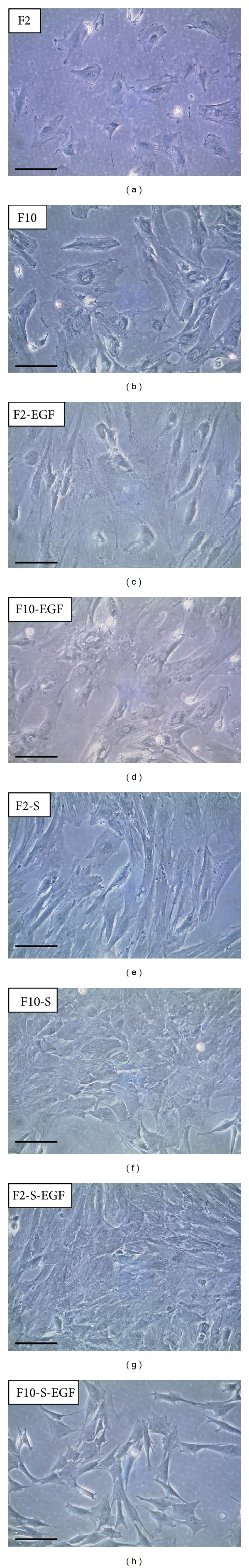
Representative pictures of oMSC in P1, cultured in media of varying composition (FCS (F), supplements (S), and epidermal growth factor (EGF)). All cells showed typical flat and spindle-shaped morphology. Ovine MSC grown in media containing EGF showed less differentiated morphology. oMSC grown in media without S and EGF showed lower density, less cell-cell contacts, and more flat and roughly shaped morphology. Scale bars 100 *μ*m.

In general, the morphology of oMSC appeared flat and fibroblast-like in all growth media. However, minor changes in morphology could be observed. Ovine MSC grown in media containing 10% FCS (F10) and/or supplements (S) were smaller with initially bigger colony size in P0. In P1, cells cultured in media containing EGF showed less differentiated morphology with more diffuse boundaries. Overall, oMSC grown in media without S and EGF showed an initially smaller colony size, less cell-cell contacts and a rough shaped morphology (Figure 1). Furthermore, cells grown in F2-S and F2-S-EGF medium rapidly formed large cell aggregates in passage numbers > 1 (pictures not shown).

### 3.2.   Cell Proliferation

**Figure 2:**
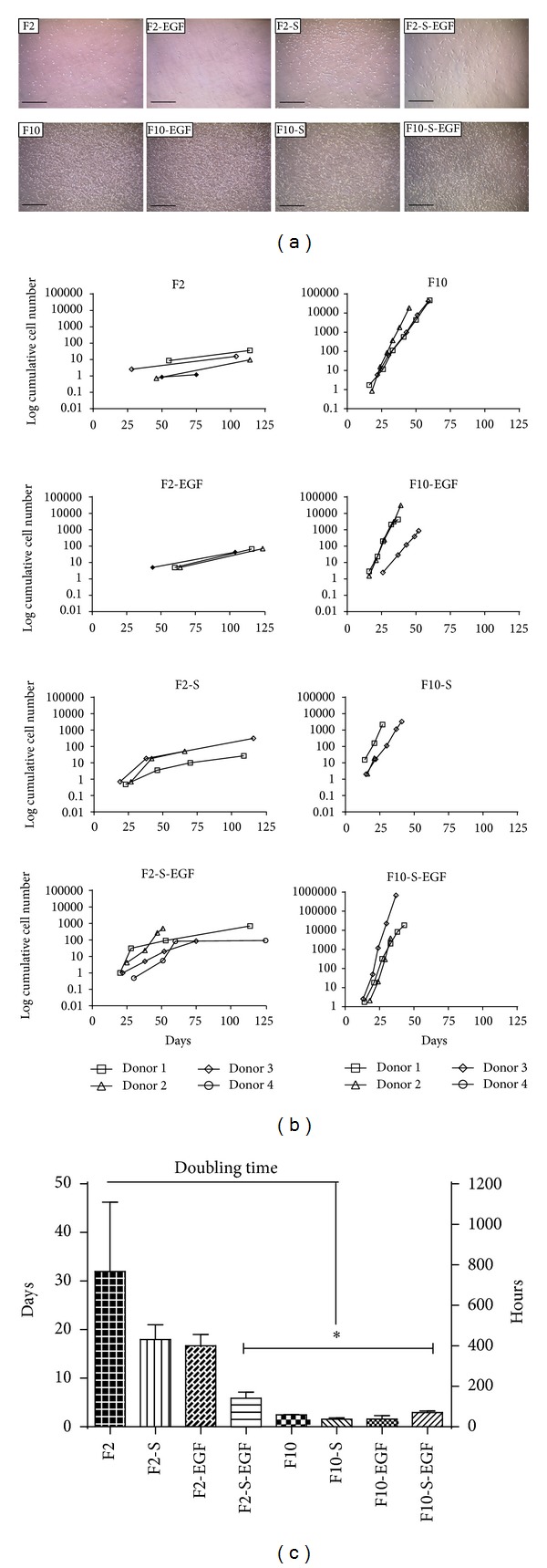
(a) Representative pictures of oMSC colony size and cell density in P0, after 9 days of culture in media of varying composition (FCS (F), supplements (S), and epidermal growth factor (EGF)). (b) Cumulative cell number of oMSC determined for up to five consecutive passages. (c) Doubling times calculated from exponential growth curves in (days) (left ordinate) or (hours) (right ordinate). Significant differences are marked with an asterisk (*P* < 0.01).

Figure 2 shows the initial colony density in P0, growth characteristics, and doubling times for each medium. oMSC cultured in media containing 10% FCS (F10) showed initially higher cell density and larger colony sizes, compared to media containing 2% FCS (F2). However, low FCS content media with addition of S (F2-S and F2-S-EGF) showed higher cell density and larger colony sizes, compared to media without S (F2 and F2-EGF).

Cells cultured in F2 and F2-EGF medium took about 60 days to pass from P0 to P1 and about 125 days to pass to P2. These media were therefore estimated as not useful for oMSC culture. The growth curves from F2-S-EGF revealed a flattened shape for two of four donors, where the proliferation decreased after passage 3. Importantly, S and EGF both decreased the doubling time in F2 medium from 31.9 ± 14.3 to 17.9 ± 5.3 (F2-S) or 16.7 ± 2.3 (F2-EGF), but the combination of these two factors decreased the doubling time significantly to only 5.9 ± 3.7. All F10 culture media promoted a rapid proliferation of oMSC with an average doubling time of approximately 2 days. For cells cultured in F10 media, S and EGF again decreased the doubling time from 2.5 ± 0.6 to 1.5 ± 0.4 (F10-S) 1.6 ± 0.3 (F10-EGF), but the combination revealed an increase in doubling time to 5.84 ± 0.4 (F10-S-EGF).

### 3.3. Surface Antigen Expression

**Figure 3:**
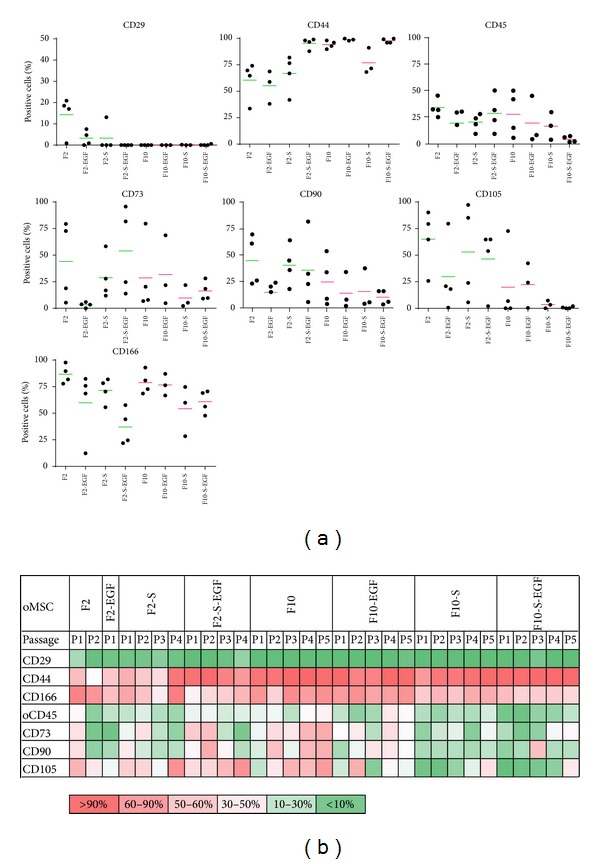
Surface epitope expression analysis of oMSC, cultured in media of varying composition (FCS (F), supplements (S), and epidermal growth factor (EGF)). (a) Percentage of positive stained cells in passage 1, detected by flow cytometry. (b) Heatmap of corresponding values of positive stained cells for up to 5 passages.

We observed large variations from donor to donor in passage 1 (Figure 3(a)), ranging from completely negative to highly positive values (CD105, F2-S-EGF: 0–70%). The heatmap in (Figure 3(b)) shows the summarized surface epitope expression for up to five passages. CD44 was consistently detected in each medium, although to a higher degree in the F10 media. CD45 was overall low, but instead, in F10-S-EGF it was even more decreased. The expression of MSC associated surface epitopes CD73, CD90, and CD105 was increased in F2 media. EGF led to a downregulation of those markers. However, in combination with S, the downregulation was decelerated. In each of the F10 media, the addition of S had a more prominent effect on the downregulation of the surface markers, while EGF alone had nearly no influence on those markers at all. Interestingly, the addition of EGF led to a downregulation of all of those markers, both in F2 and F10 media. Furthermore, the heatmap demonstrates that the addition of S in F10 media led to an overall decrease in the detection of CD45 and the hMSC associated markers CD73, CD90, and CD105. CD45 was particularly high in medium F2, where all epitopes seemed to be expressed to a higher level than in the other media. The addition of EGF and S reduced the expression of CD45 and CD166 in both, F2 and F10 media. F10 media showed overall lower expression of CD73, CD90, and CD105. Remarkably, the result did not change much with ongoing passages, especially for measurement of CD29, CD44, and CD166.

### 3.4. Differentiation in F10 and F2-S-EGF Medium

As stem cells are characterized by their ability to differentiate, we tested the response of oMSC after preculture in F2-S-EGF and F10 media to osteogenic, adipogenic, and chondrogenic stimuli. Differentiation was assayed according to published protocols for hMSC and oMSC (Table 2).

#### 3.4.1. Osteogenic Differentiation of oMSC

**Figure 4:**
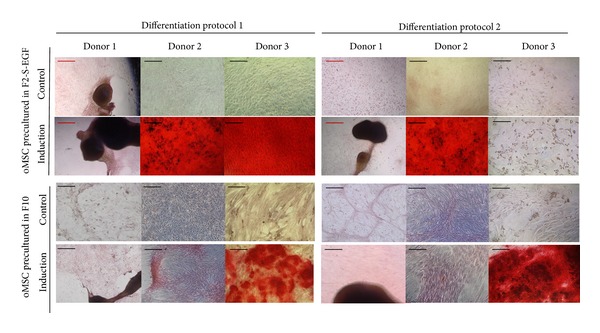
Alizarin red staining after osteogenic differentiation of oMSC precultured in F10 or F2-S-EGF medium and subsequently differentiated following two established protocols (varying in induction media and cell density) for 21 days. *n* = 3, red scale bars 500 *μ*m and black scale bars 100 *μ*m.

Figure 4 summarizes the results of osteogenic differentiation. oMSC precultured in F2-S-EGF and F10 media both showed differentiation into the osteogenic lineage for at least one of three donors. Cells of donor 1 did not show any differentiation in both protocols but a prominent formation of aggregates. Cells of donor 2 were able to differentiate towards osteoblasts using either protocol 1 or protocol 2, when precultured in F2-S-EGF medium but not when precultured in F10 medium. Instead, donor 3 could be differentiated successfully into the osteogenic lineage with protocol 1 for both, preculture in F2-S-EGF medium and F10 medium. Thus, variation in osteogenic differentiation is influenced by donor variations, protocol dependent variations, and preculture dependent variations. 

#### 3.4.2. Adipogenic Differentiation of oMSC

**Figure 5:**
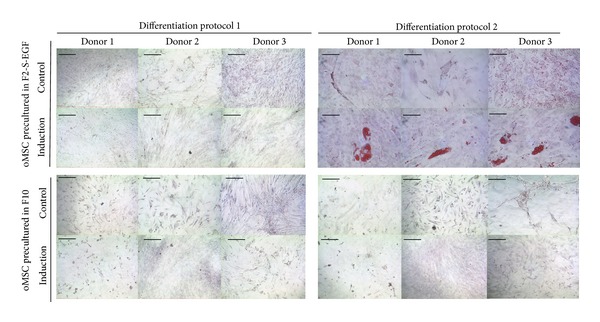
Oil red O staining after adipogenic differentiation of oMSC precultured in F10 or F2-S-EGF medium and subsequently differentiated following two different established protocols (varying induction media and cell density) for 21 days. *n* = 3. Scale bars 100 *μ*m.

Adipogenic differentiation of oMSC was performed using two different protocols (Table 3) and two preculture conditions (F2-S-EGF medium versus F10 medium). When investigating the adipogenic fate of oMSC, the presence of small lipid droplets in all control culture conditions (protocol 1 and 2, F10 and F2-S-EGF preculture) was noticeable (Figure 5). No outstanding lipid droplet formation was achieved by differentiation culture following protocol 1 or 2, when oMSC were precultured in F10 medium. However, preculture in F2-S-EGF resulted in prominent lipid droplet formation for all three donors, when using protocol 2 adipogenic differentiation (Figure 5). In contrast to the osteogenic differentiation, as each of the donors differentiated using protocol 2, we did not detect donor variations for adipogenic differentiation but a strong medium dependent differentiation.

#### 3.4.3. Chondrogenic Differentiation of oMSC

**Figure 6:**
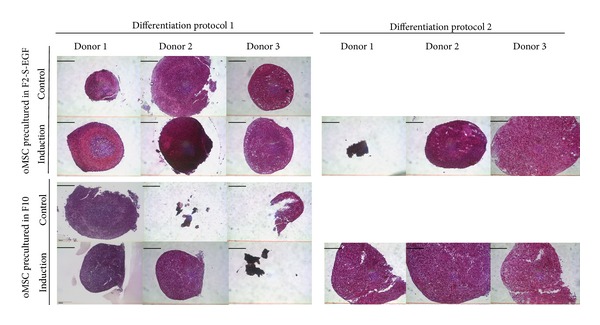
Safranin red staining after chondrogenic differentiation of oMSC precultured in F10 or F2-S-EGF medium and subsequently differentiated following two different established protocols (TGF-*β*3 or TGF-*β*1/BMP-7) for 21 days. Proteoglycans in the extracellular matrix are stained in deep red. In 2 control conditions no pellet could be obtained during culture, indicating a low overall robustness of the pellets. *n* = 3. Scale bars 200 *μ*m.

After osteogenic and adipogenic differentiation, we followed two different protocols (Table 4) to induce chondrogenic differentiation of oMSC. Chondrogenic differentiation was performed by using either TGF-*β*3 (protocol 1) or TGF-*β*1 in combination with BMP-7 (protocol 2).  Figure 6 summarizes the results for chondrogenic differentiation of oMSC. While pellets were considerably larger when cells were precultured in F10 medium and induced following protocol 2, cells precultured in F2-S-EGF medium and induced via protocol 1 showed more efficient differentiation, as indicated by pellet morphology and cell structure. In 2 control conditions no pellet could be obtained during culture, indicating a low overall robustness of the pellets. Spontaneous differentiation was also observed in the controls of all three donors, when precultured in F2-S-EGF medium. Again, we found the differentiation of oMSC to be donor dependent, protocol dependent, and preculture dependent.

## 4. Discussion

Recently, numerous studies were published employing sheep stem cells from bone marrow, referred to as ovine mesenchymal stem cells (oMSC) [19–21, 23, 33, 41, 42]. Since there is no consent on expansion (varying culture media) and characterization of oMSC (varying differentiation protocols and surface epitope analyses), we here investigated the impact of variations in culture conditions on proliferation, differentiation, and expression of surface epitopes associated with MSC. 

The oMSC culture in media of different compositions resulted in morphological variances, as previously described for hMSC and other cell types [43–45]. However, it is not clear if and how these morphological changes are related to cell functions or differentiation potential. 

The fastest colony formation and proliferation were observed in media containing 10% FCS. An increased proliferation depending on the FCS content has already been described for MSC of human and other species origin, as well as for many other cell types [46–49], underlining the importance of serum content for growth media. The addition of EGF or S (MCDB-201, dexamethasone, L-ascorbic acid, and ITS-plus) alone resulted in a reduced doubling time for both high and low serum content media, while the combination of EGF and S resulted in an even more reduced doubling time for F2 media. In F10, no further reduction of the doubling time could be achieved by the combination of these factors. It has been described for hMSC that the proliferation rate is significantly enhanced by culture with certain growth factors such as EGF, fibroblast growth factor (FGF), or platelet-derived-growth-factor- (PDGF-) BB [47, 50, 51]. However, the addition of EGF to a low serum content medium resulted in a nonfeasible culture of oMSC with more than 65 days from P0 to P1. oMSC were able to proliferate in combination with other supplements, and the doubling time was remarkably reduced in comparison to medium containing 2% FCS alone. Therefore, not only one but several factors work synergistically for fast or slow proliferation of oMSC.

In F2-EGF medium the average expression of most of the surface epitopes was at the lowest rate, while in F2 medium it was at the highest rate (Figure 3(a)). This context indicates that growth factors play a key role for the expression or depression of surface epitopes associated with stem cells. For hMSC, soluble EGF has been shown to trigger ERK and Akt/PKB signalling, which can lead to phosphorylation of surface proteins, by activation of the EGF receptor [47]. The connection between EGF and surface marker depression of oMSC still needs to be elucidated, especially when considering the foreign species origin of EGF used in this study (lack of availability of sheep growth factors). The appropriateness of human growth factors and fetal calf serum for culture of ovine cells may be worthy of discussion. Although there are studies employing sheep autologous serum for oMSC culture [17], our study aimed at comparing media with standard components frequently used in cell culture. There may be of course differences in response to autologous growth factors; nevertheless, our results add to basic knowledge on oMSC behaviour in different culture media. Since anti-human antibodies do not necessarily cross-react with ovine cells, the use of anti-human antibodies, although frequently applied in previous studies [19–21, 23], is an imperfect methodology, which further emphasizes the need for established surface markers for oMSC characterization. 

Interestingly, the surface epitope profile did not change considerably with ongoing passages. This has also been shown before for hMSC [52]. However, hMSC lose their potential to differentiate with passage 5-6, and the surface markers commonly investigated for MSC might therefore not be connected to differentiation, especially as some types of mature cells like fibroblasts exhibit the same set of MSC markers [52]. 

It is likely that the high expression of CD45 in F2 media indicates differences in isolation of cocultured cells like macrophages that are known to frequently contaminate MSC cultures [30, 53], depending on the media composition (FCS concentration). 

Taken together, the analysis of surface marker expression revealed remarkable differences depending on FCS content, media supplementation, addition of growth factors, and donor-dependent differences.

As mentioned before, there is large variation in the characterization protocols for oMSC. For surface marker expression, Rentsch et al. report positive immunofluorescence staining for CD9, CD44, CD54, CD73, CD90, CD105, and CD166, as well as successful trilineage differentiation with the afore mentioned protocol 2. The cells were previously cultured in medium, which was analogous to our F10. Our results, however, do only partly agree with those obtained by Rentsch et al., regarding the positive expression of CD73, CD90, and CD105, which we found to be inconsistent in our study. This fact may be due to the differences in surface marker detection, as well as differences in the applied antibodies (species specificity were not mentioned). 

Flow cytometry results by McCarty et al. also described oMSC to be highly positive for CD44 and CD166, but in contrast to Rentsch et al., to be negative for anti-human CD90 and CD105, which coincides with our results. In the same study, stimulatory effects on proliferation by EGF on oMSC were also confirmed and were described to be even more prominent than in media with 10% FCS only. Spontaneous adipogenic differentiation or large donor variations were not described in these studies. 

Although we did not observe a decisive impact on the differentiation, the addition of growth factors to oMSC culture should be carefully considered, as the measurement of the tested surface marker set was decreased by EGF addition. Otherwise, the FCS content of 10% without EGF led to a significant reduction in the doubling time and retainment in the expression of most of the surface markers, but it did not promote the adipogenic differentiation in our study. Considerable variations in FCS content and additions of supplements or growth factors are also usual for hMSC culture. However, standardized protocols to measure surface epitopes and perform differentiation in osteogenic, adipogenic, and chondrogenic lineage have been established [31].

Considering the various sources of influence, it is very likely that the culture conditions for oMSC from sheep bone marrow might have an influence on their response to chemical, physicochemical, or even topographical factors in tissue engineering approaches. A separation by binding of antibodies to specific surface epitopes is routinely implemented for the isolation of some cell types (hematopoietic stem cells, mouse MSC). We propose that for oMSC, donor variations might be reduced when preselection of surface epitopes [23] such as CD44 in combination with CD166 would be performed, as these markers are frequently reported to be highly positive for oMSC [19–21, 23]. Despite the overall strong expression of CD44, there is still no standard for surface epitopes that should be expressed by oMSC. Studies investigating the multipotency of oMSC vary in the selection of surface epitopes and even testing the same epitopes resulted in different outcome (anti-human markers were found to be positive and negative depending on the study) [17, 19–21, 23].

Whereas for hMSC, a loss of surface epitope expression by growth factor supplemented culture had no effect on their ability to differentiate [47]; this context still needs to be clarified for oMSC. Although it has been shown that oMSC are also able to transdifferentiate into cells with neuronal phenotype, this goal was only achieved with variable success [18]. Additionally, in the same study, large donor variations were reported. Together with the spontaneous differentiation observed in our study, it is possible that multipotency of oMSC might not be stable under recently accepted cell culture conditions. Furthermore, the only selection factor for oMSC is plastic adherence with fibroblast-like morphology. Thus, different progenitor stages might be isolated that are either inducible or noninducible by culture media [3, 54]. It has already been described for hMSC that “cells in such cultures differ markedly to the point that some have progenitor properties and multipotent differentiation capacity, whereas others are devoid of this ability and are not unlike what is commonly called fibroblasts” [3]. We here propose a similar heterogeneity for culture of oMSC. 

## 5. Conclusion

This study adds to several previous studies that demonstrated the influence of the choice of expansion media not only on growth characteristics, but also on the surface epitope phenotype and differentiation potential of MSC [55]. We here compared these characteristics for oMSC, following culture in expansion media with varying FCS content, EGF, and/or other supplementation. The oMSC behavior and phenotype is dependent on the donor, the medium of preculture, and the differentiation protocol. We therefore conclude that the conditions of oMSC culture will also affect the composition of oMSC population and therefore the outcome of oMSC-based tissue engineering strategies. Hence, a standard for oMSC culture conditions and minimal criteria for their characterization, analogous to the position papers by Horwitz et al. [29] and by Dominici et al. [30], is obligatory. Next steps should focus on eliminating protocol- and medium-related variations to receive reproducible results when using oMSC for tissue engineering studies using sheep as a large animal model.

## Figures and Tables

**Table 1:** Composition of growth media for culture of oMSC.

100 mL medium	F2	F10	F2-EGF	F10-EGF	F2-S	F10-S	F2-S-EGF	F10-S-EGF
DMEM 1 g/L glucose [mL]^1^	97	89	97	89	56.4	51.6	56.4	51.6
L-Glutamine penicillin streptomycin (LGPS) [mL]^1^	1	1	1	1	1	1	1	1

F								
FCS [mL]^2^	2	10	2	10	2	10	2	10

S								
MCDB-201 [mL]^3^	—	—	—	—	37.6	34.4	37.6	34.4
ITS + Premix 1x [mL]^3^	—	—	—	—	1	1	1	1
Dexamethasone^3^, stock 100 mM [*µ*L], final conc. 1 nM	—	—	—	—	1	1	1	1
L-Ascorbic acid^3^, stock 500 mM [mL], final conc. 100 *µ*M	—	—	—	—	2	2	2	2

EGF								
EGF [ng/mL]^3^	—	—	10	10	—	—	10	10

^1^PAA, Coelbe, Germany.

^2^Pan Biotech, Aidenbach, Germany.

^3^Sigma-Aldrich, Steinheim, Germany.

**Table 2:** Osteogenic differentiation media according to standard protocols

Osteogenic differentiation	Protocol 1 [31]	Protocol 2 [21]
DMEM 1 g/L glucose		
FCS [%]	10	10
Dexamethasone [*µ*M]	0.1	0.1
Sodium-ß-glycerophosphate [mM]	10	10
L-ascorbic acid [mM]	0.05	0.05
Cell density [/cm^2^]	31.000	2.000

**Table 3:** Adipogenic differentiation media according to standard protocols.

Adipogenic differentiation	Protocol 1 [31]	Protocol 2 [21]
Adipogenic induction medium		
DMEM 4.5 g/L glucose		
FCS [%]	10	10
Dexamethasone [*µ*M]	1	1
Indomethacin [*µ*M]	0.2	200
Insulin [mg/mL]	0.01	0.01
3-Isobutylxanthin [mM]	0.05	0.5
Cell density [/cm^2^]	80.000	30.000
Adipogenic maintenance medium		
Serum [%]	10	
Insulin [mg/mL]	0.01	

**Table 4:** Chondrogenic differentiation media according to standard protocols.

Chondrogenic differentiation	Protocol 1 [31]	Protocol 2 [19]
DMEM 1 g/L glucose		
Serum depleted		
Dexamethasone [*µ*M]	1	1
L-Ascorbic-acid-2-phosphate [mM]	0.17	0.17
Sodium pyruvate [*µ*g/mL]	100	100
Proline [mM]	0.05	0.05
ITS-Plus Premix [mL]	5	5
Human transforming growth factor-ß_3_ (TGF-ß_3_)	10 ng/mL	
Human transforming growth factor-ß_1_ (TGF-ß_1_)		10 ng/mL
Bone morphogenic protein-7 (BMP-7)		100 ng/mL
Cell density [/pellet]	1.000.000^1^	1.000.000

^1^The original cell number of 250.000 hMSC per pellet [31] was adapted to 1.000.000 oMSC per pellet, as oMSC are considerably smaller than hMSC.

**Table 5:** Antibodies used for flow cytometry analysis of oMSC to detect surface epitopes.

Antibody	Alternate name	Reactivity	Identical amino acid residues between man and sheep [%]^3^	Volume [*µ*L] per test (in 100 *µ*L PBS)
CD29^1^	Integrin beta 1	PE rabbit anti-human	93	10
CD44^2^	H-CAM, PGP-1	FITC mouse anti-sheep	—	5
CD45^1^	Leukocyte common antigen	FITC mouse anti-sheep	—	10
CD73^1^	NT5E, 5′ nucleotidase, E5NT, eNT, NT, NT5	APC mouse anti-human	81–89	3
CD90^1^	Thy-1	FITC mouse anti-human	—	3
CD105^1^	Endoglin, ENG, END, HHT1, ORW, ORW1	PE mouse anti-human	70–75	3
CD166^1^	ALCAM	PE mouse anti-human	90	10

^1^BD Biosciences, Beckton Dickinson, Franklin Lakes, USA.

^2^Serotec, Oxford, England.

^3^Protein BLAST sequence alignment, ncbi database (including transcript and splicing variants).
